# Research Status and Development Trend of Greenhouse Gas in Wetlands: A Bibliometric Visualization Analysis

**DOI:** 10.1002/ece3.70938

**Published:** 2025-02-05

**Authors:** Gege Zhu, Yan Wang, Anshu Huang, Yingying Qin

**Affiliations:** ^1^ Key Laboratory of Ecology of Rare and Endangered Species and Environmental Protection, Ministry of Education, Guangxi Key Laboratory of Landscape Resources Conservation and Sustainable Utilization in Lijiang River Basin Guangxi Normal University Guilin China; ^2^ University Engineering Research Center of Bioinformation and Genetic Improvement of Specialty Crops, Guangxi Guilin China; ^3^ Forest Resources and Ecological Environment Monitoring Center of Guangxi Zhuang Autonomous Region Nanning China

**Keywords:** bibliometrics, CiteSpace, emission reduction strategies, greenhouse gas, web of science, wetlands

## Abstract

With the intensification of global warming, wetland greenhouse gas (GHG) emissions have attracted worldwide attention. However, the scientific understanding of wetland GHGs is still limited. To gain a comprehensive and systematic understanding of the current research status and development trends in wetland GHGs. We selected 1627 papers related to wetland GHG research from the Web of Science Core Collection database and used the bibliometric visualization analysis method to reveal the annual publication, main core research forces, research hotspots, and trends in this field. The results showed that the research in this field shows a steady upward trend. United States research institutions and scholars play a key role in this field. The research on “climate change” based on three major wetland GHGs (carbon dioxide (CO_2_), methane (CH_4_), and nitrous oxide (N_2_O)) has been continuously gaining popularity. In recent years, “water” has become an emerging core topic. More and more studies have focused on enhancing wetland pollutant treatment capacity, improving wetland ecosystem productivity, maintaining water level stability, strengthening blue carbon sink function, exploring remote sensing applications in wetlands, and promoting wetland restoration to reduce GHG emissions. Furthermore, we discussed the influencing factors of the emission of CO_2_, CH_4_, and N_2_O in wetlands and summarized the potential methods to reduce GHG emissions. The findings provide scientific guidance and reference on wetland sustainable development and GHG emission reduction.

## Graphical abstract

This study provides an in‐depth analysis of the current status and development trends of research on wetland GHG, delving into the emission patterns of CO_2_, CH_4_, and N_2_O in wetlands and their underlying influencing factors. Furthermore, we summarized a series of potential strategies and specific measures to reduce GHG emissions, aiming to provide valuable references for future research on wetland GHG and promote the effective promotion and implementation of GHG reduction and management efforts.

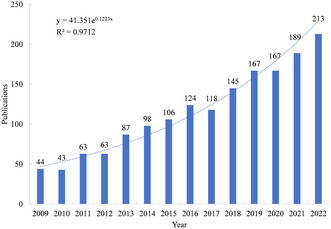

## 1 Introduction

According to the Sixth Assessment Report of the Intergovernmental Panel on Climate Change (IPCC) in 2022, the emission of GHGs climbed to the peak in human history between 2010 and 2019 (IPCC 2022; Ma et al. 2022). Climate warming has become a global problem and has aroused people's deep concern. The increasing concentration of GHGs is regarded as the main direct factor causing global warming (Ma et al. 2022). Among them, CO_2_, CH_4_, and N_2_O are recognized as the three most significant GHGs (Bhattacharjee and Chowdhury 2024). Since the beginning of the industrial era, the atmospheric concentrations of CO_2_, CH_4_, and N_2_O have increased by 40%, 150%, and 20%, respectively, compared to the preindustrial level (Tian et al. 2016), contributing up to 80% to the global greenhouse effect (Wang et al. 2022).

As an indispensable ecosystem in nature, wetlands not only have excellent primary productivity but also store about 40% of the carbon on land, which is a key component of the global carbon cycle (Ma et al. 2022). Due to the continuous growth of biomass and the continuous accumulation of organic matter in sediments, wetlands have become significant carbon sinks (Beyene et al. 2024), and at the same time, they are also crucial carbon sources (Shu et al. 2022). As wetlands occupy a special vulnerable zone between land and water, they encompass various ecological zones such as aerobic, hypoxic, and anaerobic zones. Among these, hypoxic and anaerobic zones are particularly crucial zones for GHG emissions (Liu et al. 2017). In hypoxic and anaerobic environments, the decomposition of organic matter generates significant amounts of GHGs such as CO_2_, CH_4_, and N_2_O, and their impact on global warming cannot be overlooked. Notably, despite occupying only 5%–8% of the world's land surface, wetlands contribute 20%–30% to the total emissions of the major GHGs, such as CO_2_, CH_4_, and N_2_O (Lin et al. 2022), serving as a primary natural source of GHGs (Kasak et al. 2022; Nyamadzawo et al. 2014). With the dual pressure of intensified human disturbance and global warming, it is expected that wetlands will release more GHGs and thus trigger a positive feedback loop phenomenon (Li et al. 2022), which has become one of the key issues to be addressed in global climate change research (Bao, Jia, and Xu 2023). Therefore, gaining a thorough understanding of research on the dynamics of GHGs in wetlands becomes particularly crucial, as it holds significant importance for mitigating global climate change.

At present, many studies on wetland GHGs have focused on the emission characteristics and influencing factors of GHGs in different types of wetlands. Many scholars have conducted in‐depth analyses of GHG emissions and their influencing factors (such as the carbon and nitrogen cycle process, wetland vegetation, microbial activity, water and soil conditions) in lakes, rivers, peatlands, marshes, coastal wetlands, and constructed wetlands (Camacho‐Santamans et al. 2024; Wu et al. 2024; Riquelme del Rio et al. 2024; Bartolucci and Fulweiler 2024; Martinez‐Eixarch et al. 2024; Ji et al. 2024; Jiang et al. 2024; Wang et al. 2021; Bahram et al. 2022). Although wetland GHG research has accumulated considerable results, most of them are single wetland GHG research, and there are relatively few review studies in this field. Some published review articles mainly summarized the GHG emissions and environmental driving factors of wetlands. For example, Mander et al. (2014) analyzed 158 articles to study the characteristics, mechanisms, and multiple influencing factors of GHG emissions from different types of wetlands. Hu et al. (2020) revealed the environmental drivers of GHG emissions from coastal wetlands in China based on 187 observational data. Gong et al. (2020) analyzed the impact of environmental changes on wetland GHG fluxes based on 236 data. In addition, constructed wetlands have become important review object research and practice in recent years because of their significant role in pollutant treatment, water purification, and GHG emission reduction (Jiang et al. 2023; Li, Kong, et al. 2024; Li, Zhong, et al. 2024; Xu et al. 2023; Forbrich et al. 2024). However, these review studies still lack a comprehensive understanding of the evolution of wetland GHG research, which is crucial for predicting future research trends in this field. In addition, their scope of research was narrow, and the number of papers was also small. In this study, 1627 relevant publications were analyzed for bibliometrics using CiteSpace. Through the visual analysis of annual publication volume, journals, main research forces, keywords, and other aspects, we reveal the evolution process and research status and discuss research hotspots and trends in this field. Our study aims were: (1) To explore the research status, evolution process, and frontier hotspots in the wetland GHGs field. (2) To discuss the emissions of CO_2_, CH_4_, and N_2_O from wetlands and their influencing factors. (3) To propose potential methods and specific measures for reducing GHG emissions in wetlands. This study provides valuable references and suggestions for future wetland research on GHG emissions and GHG emission reduction.

## 2 Materials and Methods

### 2.1 Data Collection

**FIGURE 1:**
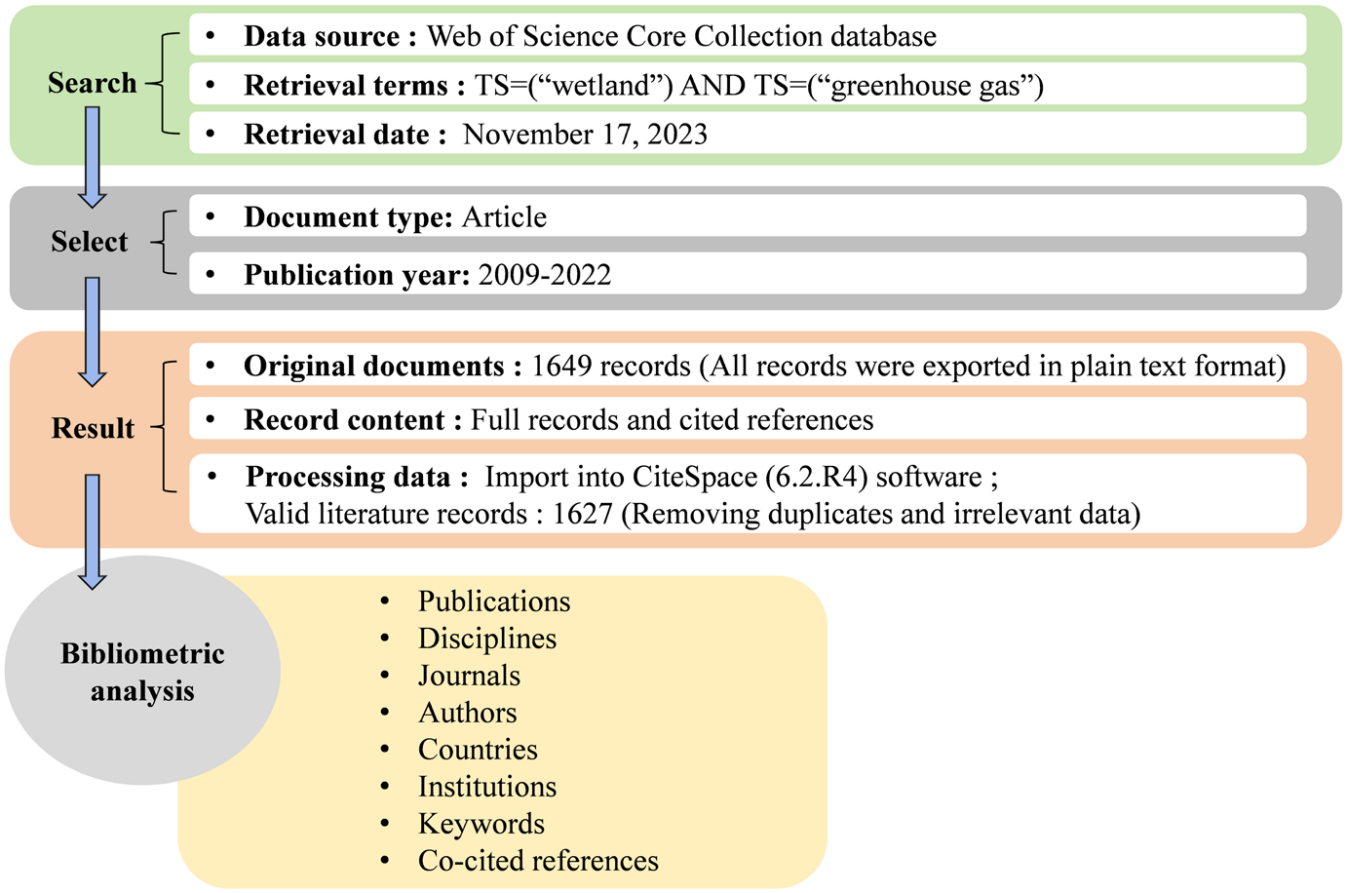
Research framework flow chart.

Web of Science is the largest and most comprehensive academic information resource repository covering an extensive spectrum of disciplines in the world, which includes prestigious scholarly journals in fields such as natural science, engineering and technology, and biomedical research. All the literature data for this study was sourced from the Web of Science Core Collection database, using the search term format TS (topic) = (“wetland”) AND TS = (“greenhouse gas”), with literature type as Article and publication year range from 2009 to 2022, a total of 1649 articles were retrieved. The retrieved data was exported in the format of complete records and reference citations in plain text files. The text data underwent conversion to the required format, and then, after removing duplicates and irrelevant data using the built‐in functions (Data/Import & Export) of CiteSpace (6.2. R4), a total of 1627 articles of wetland GHG were obtained. The data of retrieval and download for this paper is November 17, 2023. The research framework is shown in Figure 1.

### 2.2 Data Analysis

CiteSpace was used to visualize and analyze publications, disciplines, journals, authors, countries, institutions, keywords, and Co‐cited references for the 1627 articles obtained. The time span is set to 2009–2022, and the time slice is 1 year. Check the modules of node types such as countries, institutions, keywords, and disciplines for co‐occurrence network analysis. Other parameters use the system default settings. Use Excel to organize the map information data, and adjust parameters such as Threshold, Font Size, Node Size, Node Labels, Link Alpha, Node Shape, etc. to optimize the co‐occurrence map.

## 3 Results and Analysis

### 3.1 Annual Publication Volume and Trend Analysis

**FIGURE 2:**
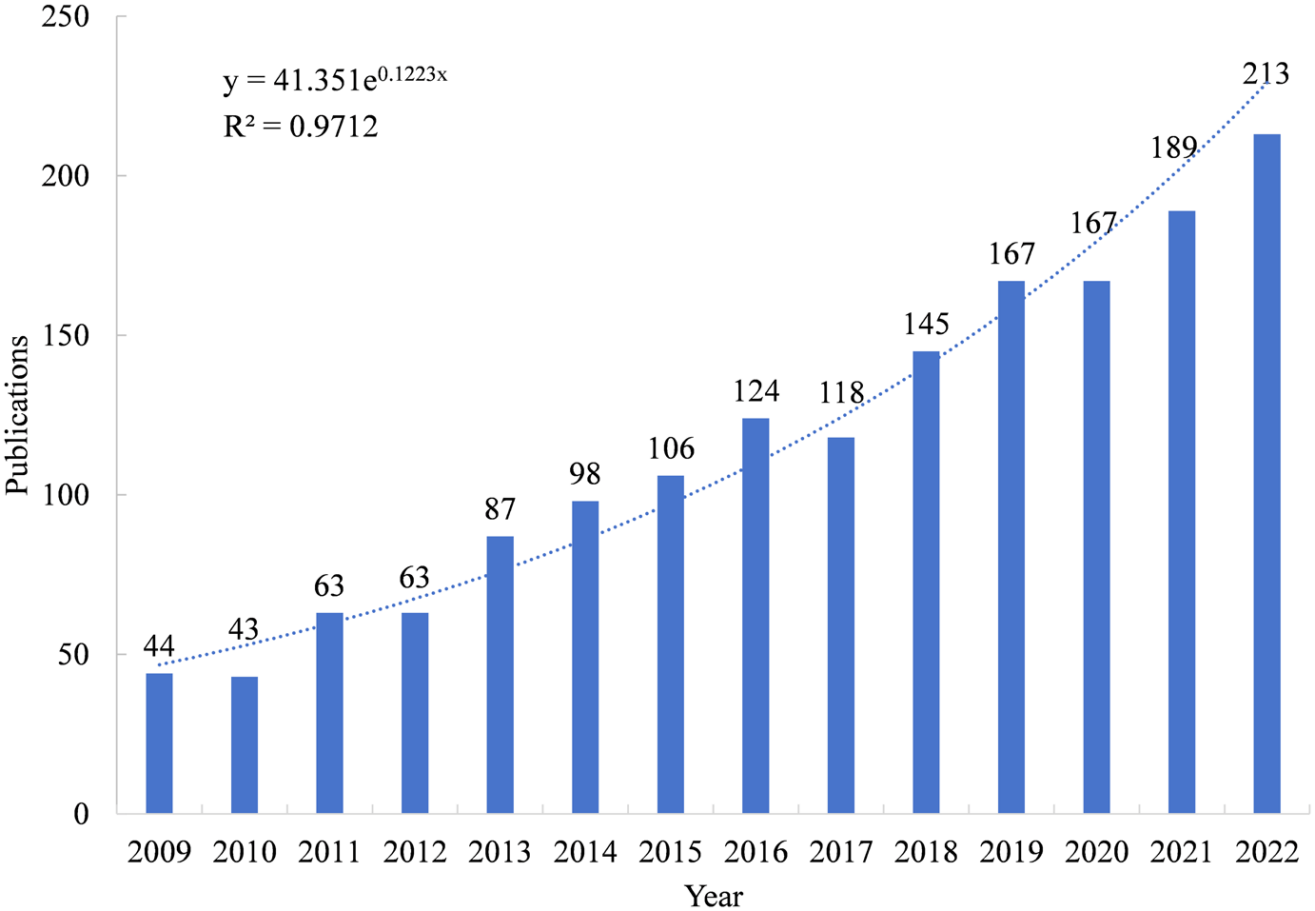
Distribution of publications from 2009 to 2022.

As shown in Figure 2, the amounts of publications in wetland GHG have exhibited a slow and steady growth trend from 2009 to 2022. Among them, the number of annual publications in the early stage of the study (2009–2014) was less than 100, while in the later period (2015–2022), it remained above 100. This implies that researchers' interest in this field has increased.

Furthermore, to predict the changes in the published articles amount on wetland GHG, we conducted a curve fitting of the published articles amount and found that it fits the exponential function model, and the coefficient of determination *R* ^2^ > 0.95 (*R* ^2^ = 0.9712), indicating that the model fitting is quite good, and it can be considered that the annual publication volume of wetland GHG follows an exponential growth pattern. Therefore, it can be concluded that wetland GHG research is currently in a rapid development stage with great potential for further development, and more new theories, methods, and technologies will continue to emerge in the future (Lin et al. 2020).

### 3.2 Discipline Category and Journal Source Analysis

After conducting a statistical analysis of the discipline categories of the 1627 wetland GHG papers, it was found that they cover a total of 78 different discipline areas. The top 10 disciplines in terms of co‐occurrence frequency are shown in Table 1. Environmental Sciences is the most frequently involved.

**TABLE 1:** Statistics of the top 10 discipline categories by co‐occurrence frequency.

Frequency	Discipline category
967	Environmental Sciences
380	Ecology
268	Geosciences, Multidisciplinary
177	Engineering, Environmental
172	Meteorology & Atmospheric Sciences
150	Soil Science
140	Water Resources
77	Biodiversity Conservation
68	Multidisciplinary Sciences
55	Marine & Freshwater Biology

Among the top 10 journals in terms of co‐cited frequency (Table 2), journals in Zone 1 account for 60%, those in Zone 2 account for 30%, and those in Zone 4 account for 10%. Among them, Global Change Biology ranks first in terms of citation frequency, with a cumulative total of 1046 times. Among the top 10 journals, papers were mainly published in environmental science and ecology journals, covering multiple disciplines such as environment, ecology, biology, soil science, meteorology and atmospheric science, and earth science.

**TABLE 2:** Distribution of top 10 journals by co‐cited frequency.

Frequency	Journals	Primary disciplines of the journal	5‐year IF
1046	*Global Change Biology*	Biodiversity Protection/Ecology/Environmental Sciences	13
942	*Nature*	Multidisciplinary Category	54.4
859	*Global Biogeochemical Cycles*	Geosciences, Multidisciplinary/Environmental Sciences/Meteorology & Atmospheric Sciences	6.6
847	*Soil Biology & Biochemistry*	Soil Science	10.4
837	*Biogeochemistry*	Environmental Sciences/Geosciences, Multidisciplinary	4.8
786	*Biogeosciences*	Ecology/Geosciences, Multidisciplinary	4.5
761	*Science of the Total Environment*	Environmental Sciences	8.6
717	*Science*	Multidisciplinary Category	50.3
664	*Journal of Geophysical Research‐Biogeosciences*	Environmental Sciences/Ecology/Geosciences, Multidisciplinary	4.1
664	*Wetlands*	Environmental Sciences/Ecology	2

### 3.3 Core Research Capability Analysis

#### 3.3.1 Author Analysis

**FIGURE 3:**
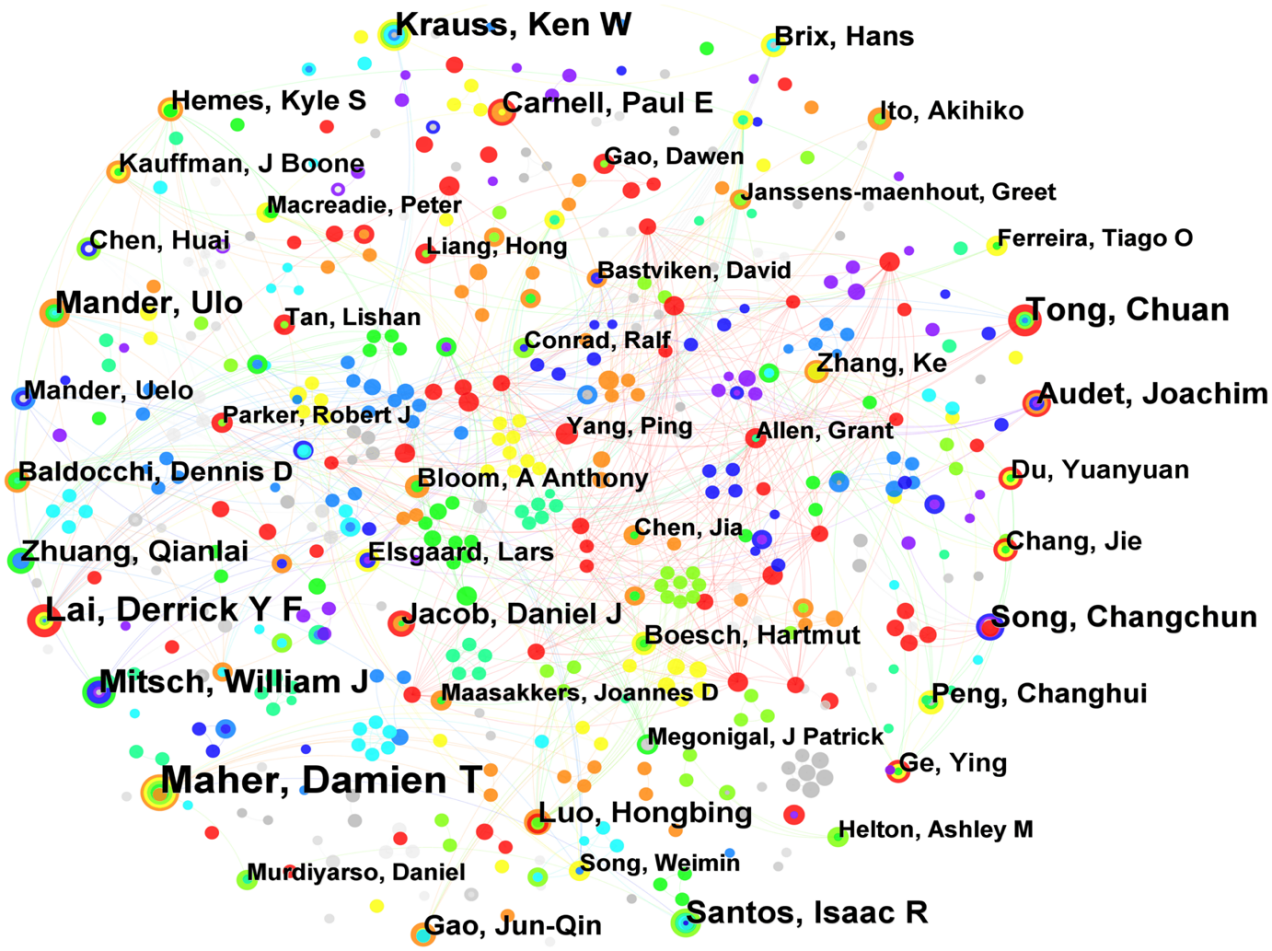
Knowledge map of author collaboration network from 2009 to 2022. The node type represents the author, and a larger node indicates a higher frequency of appearance of the author, that is., a greater number of publications. The presence of connecting lines between nodes signifies a collaborative relationship between authors.

By conducting a visual analysis of the authors publishing in wetland GHG, we obtained an author collaboration network map (Figure 3). As can be seen from Figure 3, scholars with a relatively high number of publications include Maher Damien T, Lai Derrick YF, Mitsch William J, Tong Chuan, Krauss Ken W, etc. It can also be observed that the current collaborative research on wetland GHGs is concentrated among only a small number of scholars, with the overall collaboration not being extensive or in‐depth enough. The closeness of scientific research collaboration among scholars is still very small.

#### 3.3.2 Research Country Analysis

**FIGURE 4:**
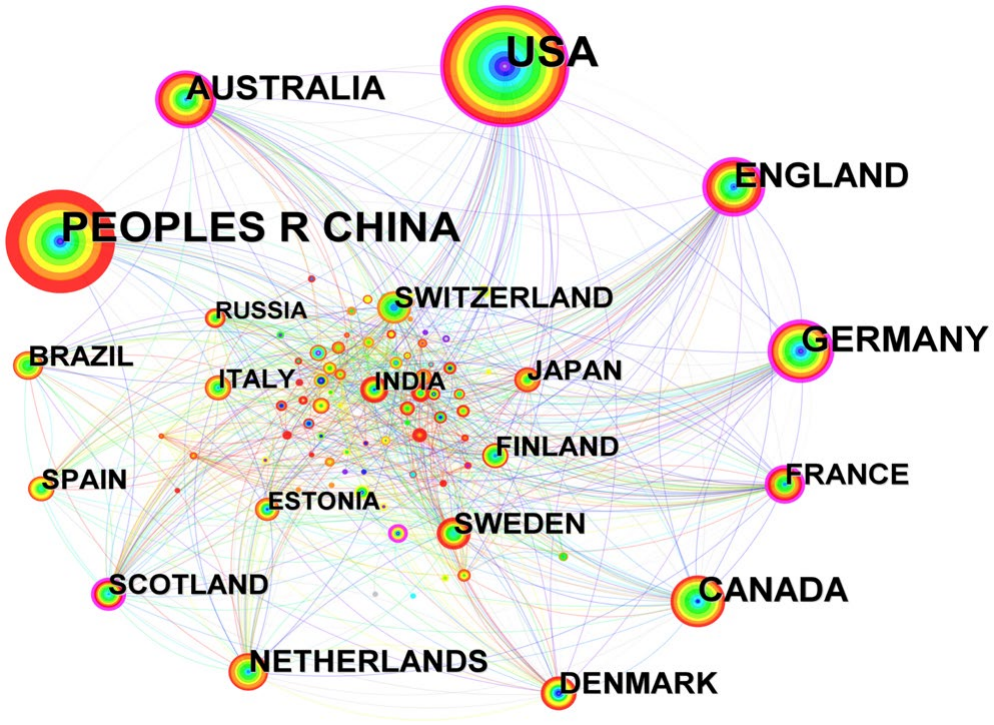
Knowledge map of country co‐occurrence network from 2009 to 2022. The nodes represent countries, and the larger the node means that countries occur more frequently; nodes with purple outer rings indicate a high centrality (centrality > 0.1). The presence of connecting lines between nodes signifies a collaborative relationship between countries.

The analysis results of the research national collaboration network are shown in Figure 4 and Table 3. As can be seen from Figure 4, the United States, Germany, England, Australia, and France have numerous connecting lines with other countries, suggesting closer collaborative relationships between these countries and others. Between 2009 and 2022, 93 countries published research literature on GHGs in wetlands, with the USA and China having the highest number of publications (Figure 4 and Table 3). This indicates that research efforts in these two countries are in‐depth and have yielded fruitful research results. Furthermore, among the top 10 countries with the highest centrality rankings, five exhibited strong centrality (> 0.1), with England being the key central node (Table 3), indicating its significant influence in this research field.

**TABLE 3:** Top 10 countries ranked by co‐occurrence frequency.

Frequency	Country	Centrality
569	USA	0.20
465	China	0.08
169	Germany	0.26
150	England	0.29
150	Canada	0.04
138	Australia	0.11
88	Netherlands	0.07
70	Denmark	0.05
67	Sweden	0.04
65	France	0.10

#### 3.3.3 Research Institute Analysis

**FIGURE 5:**
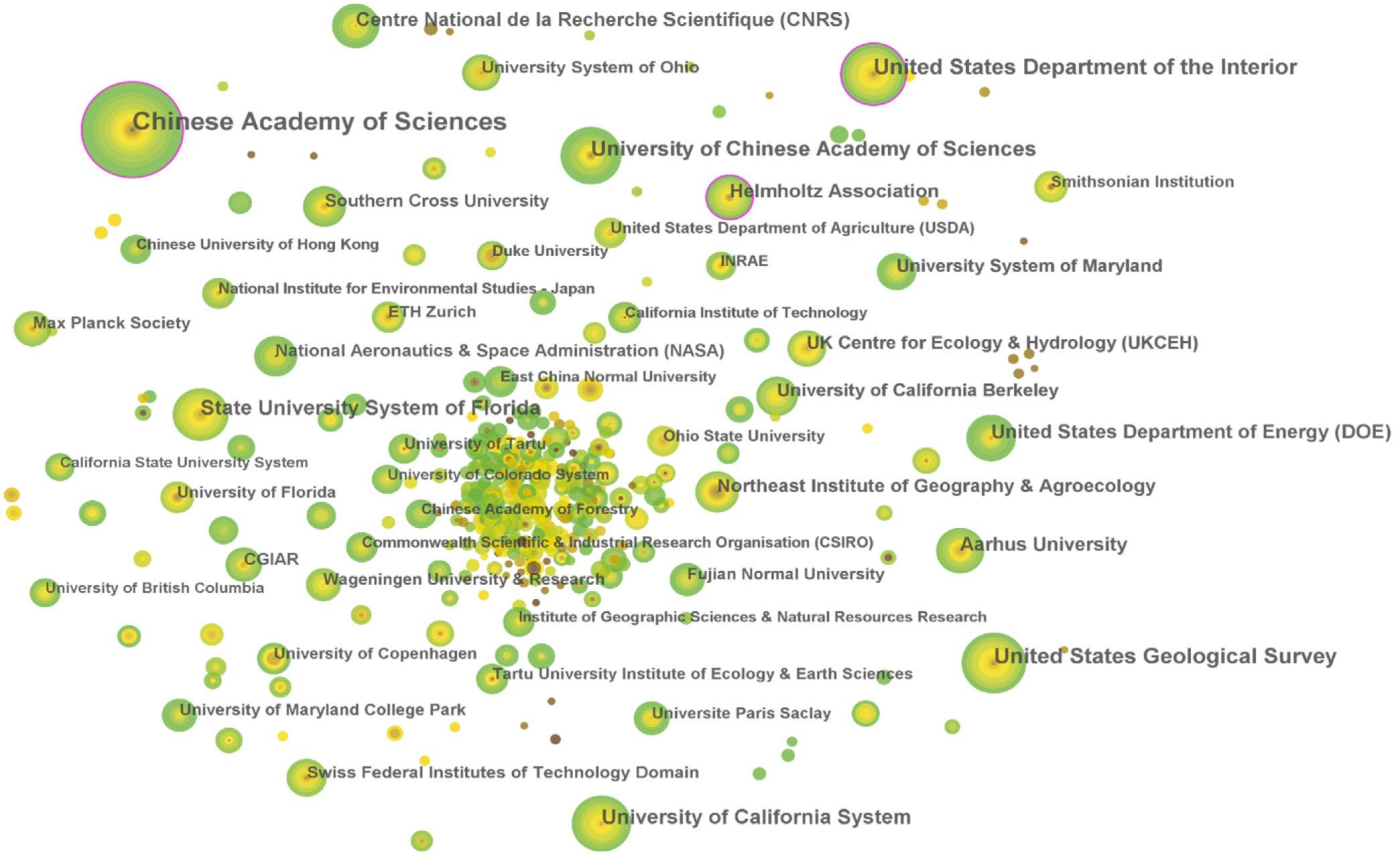
Co‐occurrence network map of publishing institutions from 2009 to 2022. The nodes represent institutions. The larger the node, the higher the frequency of occurrence and research output of the institution. Nodes with purple outer rings indicate a high centrality (centrality > 0.1).

The analysis results of the research institution collaboration network are presented in Figure 5 and Table 4. The Chinese Academy of Sciences, the United States Department of the Interior, and the United States Geological Survey have published a large number of articles and are the main institutions for global wetland GHG. In the context of global integration, the cooperation network among institutions has gradually become widespread. However, the geographical distribution of the main publishing institutions in the world is uneven, with most located in the United States and a few located in China, Denmark, and other countries. The United States is the leading force among major publishing institutions and boasts the largest contribution to the global wetland area (Gumbricht et al. 2017). Its unique geographical advantages, strong scientific research capabilities, and sustained investment in resources have propelled the United States to achieve many results in international wetland scientific research.

**TABLE 4:** Top 10 research institutions ranked by co‐occurrence frequency.

Frequency	Institution	Affiliated country	Centrality
179	Chinese Academy of Sciences	China	0.17
77	United States Department of the Interior	USA	0.11
77	United States Geological Survey	USA	0.09
66	University of Chinese Academy of Sciences	China	0.03
61	University of California System	USA	0.06
58	State University System of Florida	USA	0.09
45	United States Department of Energy (DOE)	USA	0.04
42	Aarhus University	Denmark	0.04
42	Centre National de la Recherche Scientifique (CNRS)	France	0.09
41	Helmholtz Association	Germany	0.13

### 3.4 Analysis of Research Hotspots and Trends

#### 3.4.1 Keyword Co‐Occurrence Analysis

**FIGURE 6:**
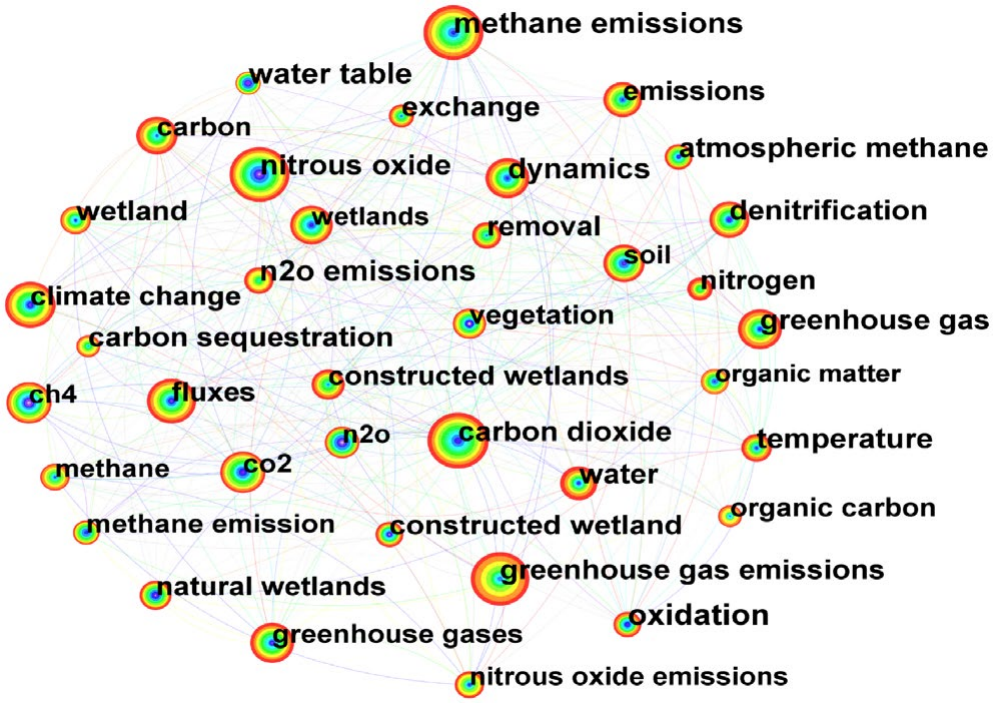
Knowledge map of keyword co‐occurrence from 2009 to 2022. The nodes represent keywords, and the larger the node, the higher the frequency of the keyword's occurrence.

The keyword co‐occurrence analysis in the wetland GHG was shown in Table 5 and Figure 6. We can see that the top five high‐frequency keywords are “carbon dioxide” (325), “methane emissions” (317), “greenhouse gas emissions” (303), “nitrogen oxides” (303), and “climate change” (212). This indicates that CO_2_, CH_4_ emissions, greenhouse gas emissions, N_2_O, and climate change are hot topics in wetland GHG. In addition, the terms “constructed wetlands” (0.07), “greenhouse gas emissions” (0.05), “oxidation” (0.05), “climate” (0.05), and “nitrogen oxides” (0.04) have high centrality. The higher the centrality of the keyword, the more significant the influence and more important the position of the keyword (Han, Peng, and Chen 2022).

**TABLE 5:** Statistical analysis of high‐frequency and high‐centrality keywords.

Frequency	High‐frequency keywords	High‐centrality keywords	Centrality
325	Carbon dioxide	Constructed wetland	0.07
317	Methane emissions	Greenhouse gas emissions	0.05
303	Greenhouse gas emissions	Oxidation	0.05
303	Nitrous oxide	Climate	0.05
212	Climate change	Nitrous oxide	0.04
203	Fluxes	Dynamics	0.04
186	Greenhouse gases	Water	0.04
181	Greenhouse gas	Temperature	0.04
180	CH_4_	N_2_O emissions	0.04
174	CO_2_	Water table	0.04
172	Dynamics	Atmospheric methane	0.04
167	Wetlands	Methane production	0.04
163	Carbon	CH_4_ emissions	0.04
159	Soil	Responses	0.04
154	Emissions	Phosphorus	0.04

The distribution of keywords in different stages is shown in Table 6. The first stage is the initial stage of wetland GHG emission assessment (2009–2013), during which basic research on GHG emissions such as N_2_O, CO_2_, and CH_4_ was initially explored, with keywords such as CO_2_, CH_4_, and GHG emissions. The second stage is the expansion stage of wetland GHG transformation, emission mechanisms, and influencing factors (2014–2018), in which the frequency of keywords significantly increased, with keywords such as soil, climate change, and dynamic changes. The third stage is the research hotspot stage of wetland GHG monitoring and emission reduction research (2019–2022), with keywords such as wetland, flux, water, climate change, and carbon. Based on these topics, we identified that scholars focused on wetland GHG fluxes, water quality status, soil physicochemical conditions, organic carbon cycle, the impact of climate change on GHGs, and the release potential and impact mechanism of wetland GHGs. These hotspots may continue to affect the study of wetland GHG emissions. In recent years, “water” has gradually become a new hotspot, and “climate change” is a hotspot that has risen steadily in the context of global warming. Furthermore, the research in this field has moved more toward the interaction between wetland GHG emissions and climate change, the carbon stoichiometry of wetland ecosystems, the synergistic effect of pollutant removal and GHG emission reduction, and the role of microbial communities.

**TABLE 6:** Distribution of high‐frequency keywords across different stages.

2009–2013		2014–2018		2019–2022	
Keywords	Frequency	Keywords	Frequency	Keywords	Frequency
Nitrous oxide	67	Carbon dioxide	138	Greenhouse gas emissions	176
Carbon dioxide	57	Methane emissions	131	Methane emissions	137
CH_4_	54	Nitrous oxide	121	Carbon dioxide	130
Methane emissions	49	Greenhouse gas emissions	94	Nitrous oxide	115
Greenhouse gases	43	Climate change	83	Fluxes	94
Soil	37	Fluxes	74	Climate change	92
Climate change	37	Dynamics	72	Greenhouse gas	88
CO_2_	36	Wetlands	71	Greenhouse gases	77
Fluxes	35	CO_2_	70	Dynamics	74
N_2_O	35	Greenhouse gases	66	Carbon	74
Greenhouse gas emissions	33	Carbon	63	Emissions	68
Greenhouse gas	31	Greenhouse gas	62	CO_2_	68
Denitrification	31	Soil	62	Wetlands	66
Wetlands	30	CH_4_	61	Water	65
Emissions	28	Emissions	58	CH_4_	65

#### 3.4.2 Co‐Cited Reference Analysis

**FIGURE 7:**
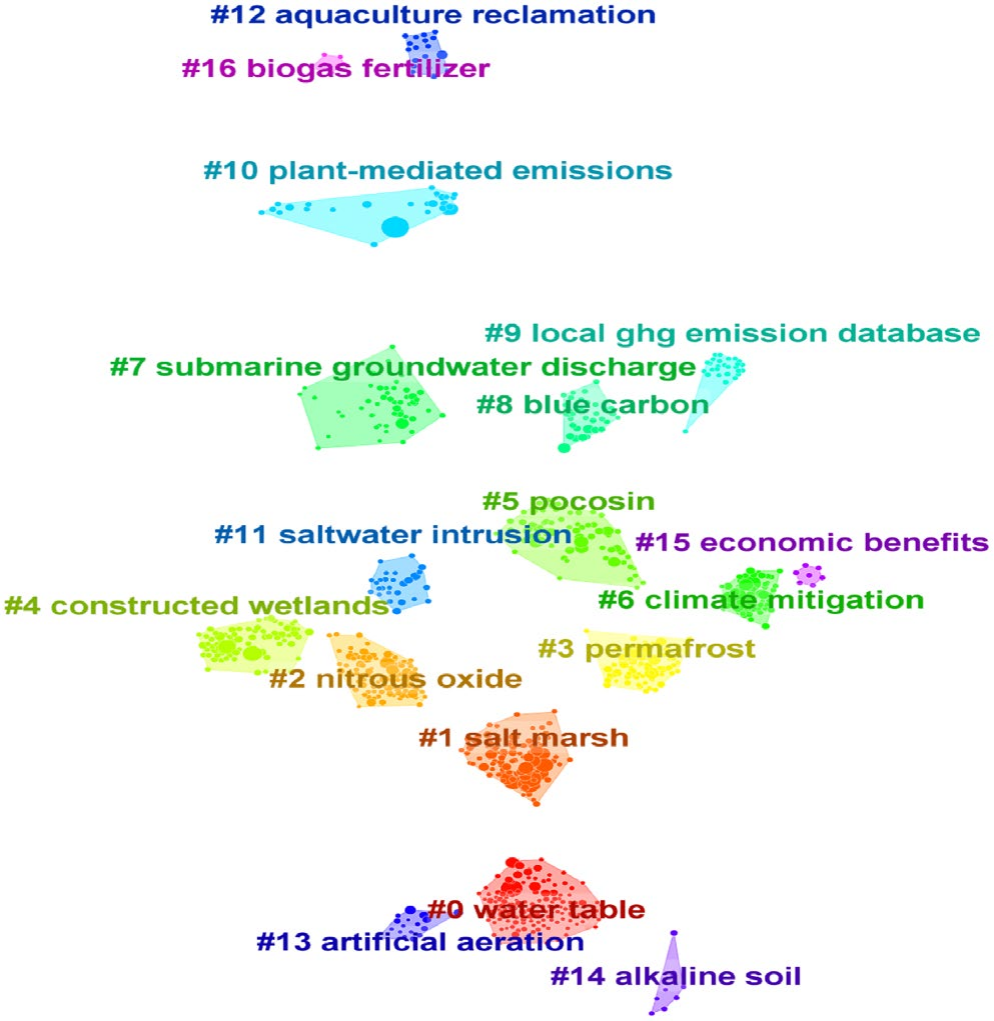
Co‐cited cluster analysis of references.

Co‐cited refers to the phenomenon where two academic papers are cited by the same paper, which reflects its influence and the degree of attention it receives. The co‐citation clustering knowledge map of the co‐citation analysis of the obtained wetland GHG literature is shown in Figure 7. The clustering network module value *Q* = 0.7513 > 0.3 and the weighted average silhouette value *S* = 0.8659 > 0.5 indicate that the clustering results are reasonable and reliable. A total of 16 clusters are generated.

**FIGURE 8:**
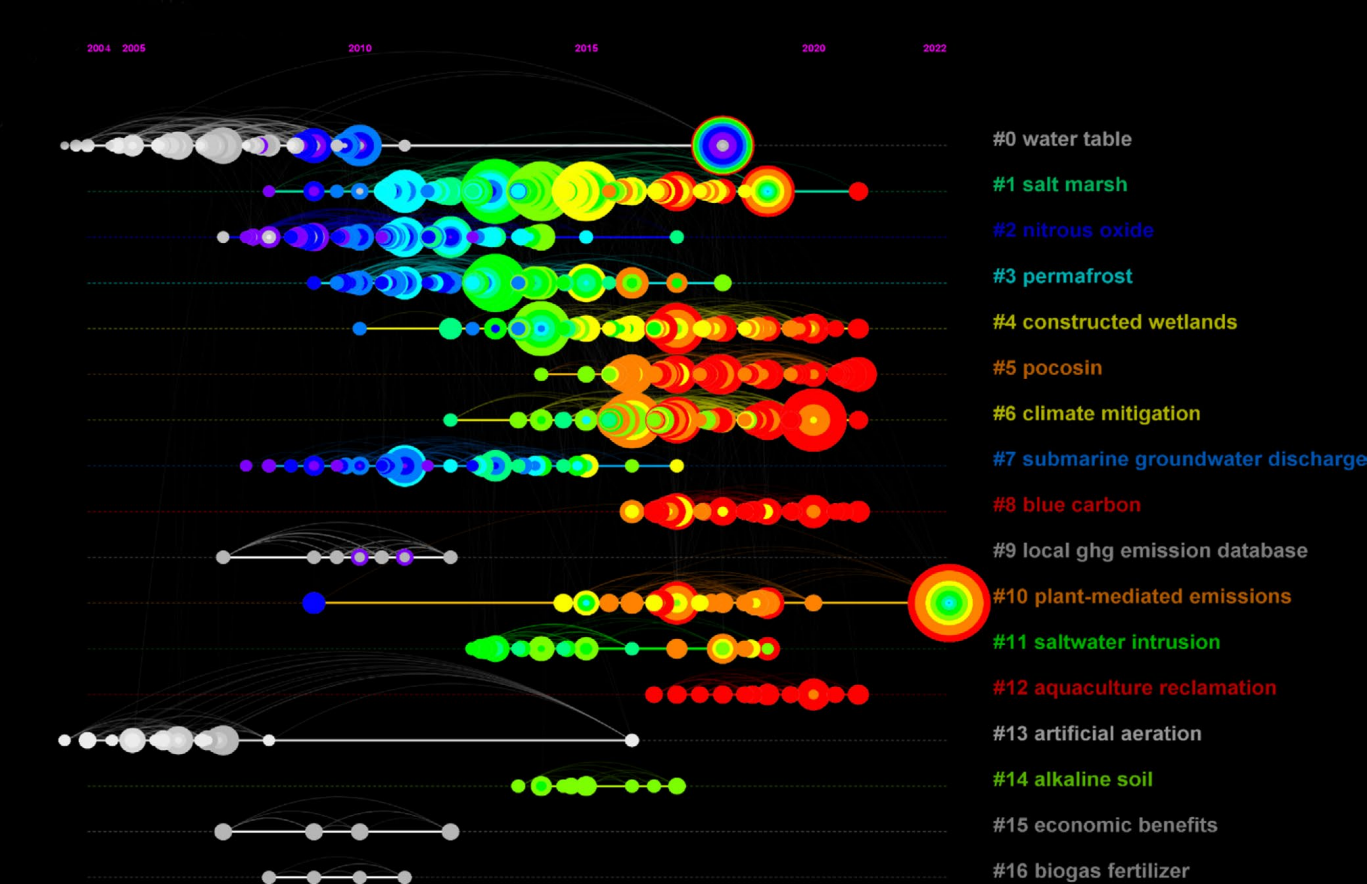
Timeline visualization of co‐cited references. The larger nodes in the map indicate a higher citation count for the corresponding document.

By converting the co‐cited clustering of papers into a timeline map, it becomes visible that documents sharing the same clustering topic are arranged chronologically along the same horizontal line (Figure 8). From the map, the duration of each cluster varies. The clusters that remain active are: #1 salt marsh, #4 constructed wetlands, #5 pocosin, #6 climate mitigation, #8 blue carbon, #10 plant‐mediated emission, and #12 aquaculture reclamation. Among them, the active time of #1, #4, and #10 is relatively long. The following is a brief analysis of the details of the above clusters.

The main topic terms in cluster #1 include greenhouse gas, climate warming, saltwater intrusion, and other related topics. This group of papers focuses on the relationship between salt marsh wetlands and GHGs, emphasizing the crucial role of salt marsh wetlands in maintaining the global carbon balance and mitigating climate change (Bridgham et al. 2013; Emery and Fulweiler 2014; Chen et al. 2013).

The main topic terms in cluster #4 include microbial community, contaminant removal, carbon source substrates, enhanced adsorption substrates, and other related topics. This group of papers mainly focuses on studying GHG fluxes in constructed wetlands. By creating and optimizing constructed wetlands, people explore substrates with strong adsorption properties to improve the wastewater treatment capacity (Vymazal, Zhao, and Mander 2021). At the same time, they regulate the microbial community structure and enhance its role in the biogeochemical cycle of carbon and nitrogen, thereby effectively reducing carbon emissions (Vymazal, Zhao, and Mander 2021; Bonetti et al. 2021; Taseli 2020).

The main topic terms in cluster #10 include *Fraxinus nigra*, soil carbon, greenhouse gas emissions, and other related topics. This group of papers explores how plants directly or indirectly influence wetland GHG emissions through physiological and ecological processes, mainly related to wetland plant ecosystem productivity research. The mediation mechanisms involve the interaction of root activity, leaf photosynthesis, and soil microbial communities (Toczydlowski et al. 2020; Feng, He, et al. 2022; Feng, Wang, et al. 2022; Gu et al. 2022; Shoemaker, Varner, and Schrag 2012).

The main topic terms in cluster #5 include carbon emission, land management, synthetic aperture radar, and other related topics. This group of papers emphasizes research on scientific regulation of wetland water levels, maintaining appropriate and stable water levels, optimizing wetland management strategies, and severely punishing illegal sewage and destruction behaviors, with a profound impact on effectively mitigating carbon emissions (Kluber et al. 2014; Gutenberg et al. 2019). In addition, research on remote sensing technology, such as synthetic aperture radar, is also discussed (Richard Allen, Wang, and Gore 2013). These measures are conducive to promoting wetland ecological restoration and protection.

The main topic terms in cluster #6 include tropospheric oxidative capacity, inverse model, spatiotemporal variations, emission inventory, and other related topics. This group of papers mainly investigates the influencing factors of tropospheric oxidative capacity, the compilation and aggregation of emission inventories, the spatiotemporal variations of gas concentrations, and the application of remote sensing techniques such as inversion models (Pitt et al. 2022; Polson et al. 2011; Morimoto et al. 2021). The aim is to understand GHG emission patterns and concentration levels, providing guiding evidence for developing effective climate mitigation strategies and promoting low‐carbon development.

The main topic terms in cluster #8 include coastal wetlands, inorganic carbon, carbon stocks, and other related topics. This group of papers focuses on blue carbon, a type of carbon characterized by its large sequestration capacity, high efficiency, and long storage time, signifying its immense potential in mitigating climate change (Zhao et al. 2022; Sheehan et al. 2019).

The main topic terms in cluster #12 include nitrogen cycling, land coverage change, methane production, and other related topics. This group of papers primarily investigates the impact of aquaculture land reclamation on GHG emissions. The aquaculture process increases emissions of CH_4_ and N_2_O, while land reclamation releases organic carbon from vegetation and soil into the atmosphere, causing a rise in GHGs (Chanda et al. 2019; Yang, Lai, et al. 2022; Yang, Tang, et al. 2022; Lin and Lin 2022). Therefore, it is necessary to adopt measures to reduce GHG emissions from aquaculture land reclamation and promote sustainable development.

## 4 Discussion

### 4.1 CO_2_ and CH_4_ Emissions in Wetlands and Their Influencing Factors

Wetlands occupy only 5%–8% of the Earth's surface area, yet they contribute 20%–30% of the global soil carbon storage (He, Ding, et al. 2024; He, Ye, et al. 2024; Xia et al. 2024). They serve as pivotal regulators and contributors to the carbon cycle and storage process in the global ecosystem (Li et al. 2022). Carbon is one of the significant factors influencing global climate change, primarily existing in the atmosphere as CO_2_ and CH_4_, which are two crucial carbon‐based GHGs contributing to global warming (Skytt, Nielsen, and Jonsson 2020; Cael and Goodwin 2023; Liang et al. 2023). The wetland carbon cycle is closely related to the biological cycle. Primary producers produce organic matter, leading to biomass accumulation (Zhou, Zhou, and Jia 2009). This biomass is then decomposed by microorganisms through the food chain, returning to the atmosphere as CO_2_ or remaining in the soil as other forms of organic matter (Zhou, Zhou, and Jia 2009). In wetlands, CO_2_ emissions mainly originate from the respiration of plants, animals, and microorganisms (Datry et al. 2018). CH_4_ is primarily produced by methanogens in wetlands, whose growth and metabolic activities are jointly promoted by the eutrophication environment, low‐oxygen conditions, and an abundant supply of plant organic substrates in wetlands, thereby driving the production and emissions of CH_4_ (Mondal et al. 2022; Venturi et al. 2021; Xu et al. 2019; Zhang et al. 2018).

The emission of CO_2_ and CH_4_ in wetlands is a relatively complex process, directly influenced or indirectly regulated by various factors such as hydrological conditions, land‐use patterns, and vegetation. Wetland hydrological flow patterns are a factor that regulates the differences in GHG emissions between adjacent wetlands. CH_4_ emissions are highest at low water flow velocities, while CO_2_ emissions peak at moderate water flow velocities (Keating et al. 2016). Rising water levels can weaken the carbon sink of wetlands, such as when the transition from medium to high water levels occurs, and sedge communities and reed communities also shift from being carbon sinks to carbon sources (Wang, Deng, et al. 2024; Wang, Fu, et al. 2024; Wang, Zhou, et al. 2024). In addition, previous studies have determined that the deeper groundwater level is conducive to CO_2_ emission, and the shallower groundwater level is conducive to CH_4_ emission in wetlands (Evans et al. 2021). Land use change will lead to the degradation of natural wetlands and affect GHG emissions. Research indicates that while artificial wetlands serve as CO_2_ sinks, their CO_2_ sequestration capacity is lower than that of natural wetlands (Zhong et al. 2016). The degradation of organic pollutants in artificial wetlands can lead to significant emissions of CO_2_ and CH_4_ (Salimi, Almuktar, and Scholz 2021), with emissions being 2–10 times higher than those in natural wetlands (Lu et al. 2018). When natural wetlands are converted into aquaculture ponds, CH_4_ emissions significantly increase, and net CO_2_ absorption decreases (Tan et al. 2020; Yang et al. 2017; Yang, Lai, et al. 2022; Yang, Tang, et al. 2022), while when peat wetlands are converted into pastures, CH_4_ emissions also increase (Tan et al. 2020). Furthermore, plants, as a crucial component of wetland ecosystems, play a pivotal role in regulating the emissions of CO_2_ and CH_4_. On the one hand, wetland plants indirectly affect CO_2_ emissions by changing environmental variables such as pH, temperature, dissolved oxygen, and carbon source availability (Barbera et al. 2015). On the other hand, the aerenchyma tissue of wetland plants serves as a transmission medium for CH_4_ exchange with the external environment. This tissue facilitates CH_4_ to bypass the oxidation zone in the surface soil layer, thereby increasing CH_4_ emissions. Simultaneously, it mediates the transport of O_2_ to the rhizosphere, where CH_4_ can be oxidized, thus reducing CH_4_ emissions (Iqbal et al. 2021). It is worth noting that invading exotic plant species, such as mangroves and *Spartina alterniflora* , enhance CO_2_ and CH_4_ emissions in coastal wetlands. It is probably associated with their higher biomass production, ventilated tissues, and efficient gas transport (Chen, Chen, and Ye 2015; Tong et al. 2012).

### 4.2 N_2_O Emissions From Wetlands and Its Influencing Factors

N_2_O, a potent GHG, possesses a greenhouse effect that is 265 times greater than CO_2_ despite its atmospheric concentration being substantially lower than that of CO_2_ (Xu et al. 2017; Bahram et al. 2022). Wetlands are significant sources of N_2_O emissions, contributing more than 10% of the total global N_2_O emissions annually (Schuster et al. 2024). N_2_O in wetlands is mainly produced by microbial‐mediated nitrification and denitrification processes and is emitted into the atmosphere through diffusion and plant transport (Rosentreter et al. 2021; Yang et al. 2021; Du et al. 2021).

N_2_O emission is closely related to wetland types, particularly coastal wetlands, which have emerged as a significant source of N_2_O emissions (Hu et al. 2020). In peatlands, if the soil maintains a consistently moist condition, the peat will absorb CO_2_ emitted by plants and accumulate organic carbon in the soil (Pärn et al. 2024). However, when the soil moisture in peatlands is at moderate or lower levels, they instead release N_2_O (Pärn et al. 2024). It is worth noting that compared with natural wetland ecosystems such as coastal wetlands, swamps, and peatlands, the N_2_O emissions from artificial wetlands are often higher, and the N_2_O flux in agricultural wetlands is often twice that of natural wetlands (Søvik and Kløve 2007; Ashiq et al. 2022). The wetland hydrology also has a significant impact on N_2_O emissions. When soil organic carbon and inorganic nitrogen contents are sufficient, soil water‐filled porosity controls the emission of N_2_O, and a decrease in water level promotes increased N_2_O emissions (Kachenchart et al. 2012). The soil of natural wetlands without water accumulation is the main area for N_2_O emissions, while the N_2_O flux of water‐logged soil is relatively small or negative (Ashiq et al. 2022). Beyond that, wetland plant growth indirectly affects the denitrification process by influencing the availability of soil moisture, mineral nitrogen, and organic carbon, thereby influencing N_2_O emissions (Rummel et al. 2021). Vegetated tidal flats typically have higher N_2_O emissions than mudflats (Xu et al. 2014), as different vegetation types have differential effects on soil aeration and microbial activities, leading to spatial heterogeneity in N_2_O emissions. In addition, physical–chemical properties such as soil moisture, temperature, C/N ratio, electrical conductivity, and pH value can also directly affect the production and emission rates of N_2_O (Feng, He, et al. 2022; Feng, Wang, et al. 2022; Lu and Xu 2014).

### 4.3 Potential Approaches for Mitigating GHG Emissions From Wetlands

Research has shown that N_2_O emissions are regulated by the depth of the average groundwater level, and deeper groundwater levels are beneficial for N_2_O emissions (Tiemeyer et al. 2016). Therefore, scientific and rational water resource management, maintaining appropriate water levels, and avoiding excessive water accumulation or drought are key measures to reduce GHG emissions such as N_2_O (Evans et al. 2021). The emission of GHGs in wetlands is also significantly influenced by wetland plants (such as *Phragmites australis* , *Suaeda salsa*, *Spartina alterniflora* and *Tamarix chinensis* ) (Goncharova et al. 2019). The oxygen secreted by plant roots can promote the oxidation of CH_4_ and reduce CH_4_ emissions (Gu et al. 2022; Xie et al. 2023). Furthermore, it is reported that the carbon sequestration of wetland plants helps reduce the total amount of GHGs emitted from wetlands into the atmosphere (Gu et al. 2022). Wetland plants absorb CO_2_ from the atmosphere through photosynthesis and convert it into organic matter, thereby achieving carbon sequestration. There are differences in the carbon sequestration abilities of different plants. For instance, the carbon sequestration ability of *Spartina alterniflora* is 2.6 times that of *Suaeda salsa* and 8.8 times that of *Phragmites australis* (Li, Kong, et al. 2024; Li, Zhong, et al. 2024). Therefore, we can enhance wetland carbon sequestration by selecting plant species with stronger carbon fixation abilities. Further, we can adopt several measures, such as constructing a wetland plant ecotone; removing dead biomass through above‐ground harvesting to effectively restore plant vitality and promote next year's plant growth; conversion of plant biomass (biomass fuels, fertilizers) into inert biochar using sustainable energy technologies to achieve more durable and efficient carbon sequestration (He, Ding, et al. 2024; He, Ye, et al. 2024; Wang, Deng, et al. 2024; Wang, Fu, et al. 2024; Wang, Zhou, et al. 2024).

Besides hydrological conditions and vegetation management, the regulation of C/N ratio in wetlands and the selection of substrate types in artificial wetlands are also effective means to reduce GHG emissions. Low and medium C/N ratios are beneficial for reducing GHG emissions, while substrates such as biochar, zeolite, and pyrite are more conducive to reducing N_2_O emissions compared to traditional gravel substrates (Huo et al. 2022). In particular, the combination of walnut shells and manganese substrates can enhance nitrification and denitrification processes, resulting in minimal N_2_O flux (Xu et al. 2021).

In summary, reducing GHG emissions from wetlands requires a comprehensive consideration of hydrological condition management, wetland plant selection and management, as well as optimization of C/N ratio and substrate type. The implementation of these measures will help maintain the health and stability of wetland ecosystems, promote the dynamic balance of the carbon cycle, and make positive contributions to addressing global climate change.

## 5 Conclusion and Prospect

This study conducted a comprehensive visual analysis of the research literature on wetland GHGs from 2009 to 2022 and discussed the research hotspots and trends in this field. The study found that the United States has made a greater contribution to this field, published the most papers, is the leader of the current major publishing institutions, and the emergence of high‐impact American scholars represented by Krauss Ken W. and Mitsch William J. Over the past 13 years, wetland GHG research can be divided into three stages, with climate change being the core issue throughout these stages, and the frequency of its occurrence is continuously increasing. In recent years, “water” has become a new research hotspot. The water quality of wetlands, wastewater treatment, and the ecological function of wetland water may inject new vitality and direction into wetland GHG research. It is worth noting that we may reduce GHG emissions by optimizing the carbon storage capacity of wetlands, such as enhancing the ability of constructed wetlands to purify sewage, maintaining the stability of wetland water levels, improving the productivity of wetland ecosystems, and promoting wetland protection and restoration. With wetlands being significant GHG sources and carbon sinks, their protection and restoration are urgent, demanding future research into adaptive measures. Consequently, it is imperative to prioritize the following crucial perspectives in future research endeavors to scientifically evaluate, mitigate, and potentially balance these GHG emissions. In the future, we can explore in depth these aspects:

### 5.1 Wetland Degradation and GHG Emissions

Wetland degradation causes serious problems, including the deterioration of wetland carbon storage capacity and the intensification of GHG emissions (Cui et al. 2022; Lin et al. 2021; Xu et al. 2019). It is necessary to expand research on rewetting restoration and vegetation restoration. In terms of methodology, encompassing the establishment of water replenishment systems, the utilization of surface and subsurface flow wetland processes to enhance self‐purification capabilities, as well as the cultivation of local vegetation and the management of exotic species. This would benefit scholars by developing more targeted wetland restoration and emission reduction measures.

### 5.2 The Application of Wetland GHG Remote Sensing

Although remote sensing technology's data resolution has significantly improved, it is still necessary to cope with the challenges of wetlands' complex spectral characteristics. Even if the combination of hyperspectral, multi‐spectral, and radar data can reduce spectral loss, problems such as spatial registration changes and radiation mismatch will also interfere with it (Yuan et al. 2022). At the same time, data acquisition and processing are costly, time‐consuming, and vulnerable to weather. Therefore, to address these challenges, future research directions may focus on optimizing spatial registration algorithms for remote sensing data and improving the accuracy of radiometric correction (such as exploring spatial registration algorithms using deep learning techniques and improving solar radiation correction methods for remote sensing images); developing a remote sensing monitoring platform that adapts to different weather conditions and exploring more efficient data acquisition methods (such as using Unmanned Aerial Vehicle remote sensing technology, intelligent data processing algorithms, and cloud computing platforms).

### 5.3 The Application of the Wetland GHG Model

The wetland GHG emission model serves as a robust instrument in elucidating the intricacies of the wetland carbon cycle and assessing GHG emissions. Combining vision transformer deep learning technology and multi‐spectral satellite imagery enables automated, high‐resolution CH_4_ emissions detection worldwide (Rouet‐Leduc and Hulbert 2024). Integrating remote sensing vegetation cover classification into the earth system model also significantly improves the accuracy of CH_4_ emission assessment (Yazbeck et al. 2024). However, the accuracy of multi‐spectral satellite images is limited when capturing surface information. Moreover, the wetland water level changes daily, and the vegetation coverage is deeply affected by the wetland water depth. Although the earth system model can predict the water dynamics of wetlands, its resolution and accuracy need to be improved. In addition, there is a lack of effective integration of microorganisms and models, ignoring the key influencing factors at the microbial level. Therefore, exploring the integration of microbial processes and transport mechanisms into the model framework while improving the accuracy of model validation data sets (such as remote sensing and ground monitoring data) can better serve GHG emission assessment.

### 5.4 The Development and Implementation of Wetland GHG Emission Reduction Technologies and Methods

Microplastics are a focused environmental concern, and they can enter wetland ecosystems through different routes. Particularly, microplastics in wetlands not only disrupt the carbon and nitrogen cycle as exogenous carbon sources but also have the potential to exacerbate GHG emissions (Li, Kong, et al. 2024; Li, Zhong, et al. 2024). The research on microplastics is more about the influence of their type, size, and concentration on the carbon and nitrogen cycle mechanisms of wetlands (Ibrahim et al. 2021; Li, Kong, et al. 2024; Li, Zhong, et al. 2024). In addition, some scholars have also paid attention to the effect of microplastics on wetland microbial community structure (Wang, Deng, et al. 2024; Wang, Fu, et al. 2024; Wang, Zhou, et al. 2024). Given this, we can further explore the potential of wetland microorganisms in degrading microplastics on this basis. Future research can focus on screening, cultivating, and applying these microorganisms with microplastic degradation functions, aiming to develop more efficient and environmentally friendly wetland microplastic degradation technologies.

### 5.5 Global Warming and GHG Emissions From Wetlands

Research has revealed that with a rise in average temperature by 1.5°C–2°C, the warming potential of wetlands escalates by 57%, triggering a surge in CO_2_, CH_4_, and N_2_O (Bao, Jia, and Xu 2023). Confronting the grave challenge of escalating global temperatures, this trend not only disrupts the balance between wetland carbon storage and GHG emissions but also further aggravates the vicious cycle of global climate change. Therefore, urgent and effective measures must be implemented to mitigate this trend. These include, but are not limited to, establishing nature reserves, stringently regulating wetland development and destruction, establishing wetland carbon sink monitoring stations, and regularly collecting and conducting in‐depth analyses of GHG emission fluxes. Additionally, steps must be taken to reduce the surface temperature of wetlands, enhance the coverage of wetland vegetation, and minimize the proportion of bare soil on the wetland surface, aiming to decelerate the trend of climate warming.

## Author Contributions

**Yingying Qin:** conceptualization (lead), funding acquisition (lead), writing – review and editing (lead). **Gege Zhu:** methodology (lead), software (lead), visualization (lead), writing – original draft (lead). **Yan Wang:** data curation (equal), investigation (equal). **Anshu Huang:** data curation (equal), methodology (equal), resources (equal).

## Conflicts of Interest

The authors declare no conflicts of interest.

## Supporting information


Data S1.

Data S2.

Data S3.

Data S4.

## Data Availability

I have shared my data in the Supporting Information (Data [Link], [Link]).
